# Mercury Exposure from Domestic and Imported Estuarine and Marine Fish in the U.S. Seafood Market

**DOI:** 10.1289/ehp.9377

**Published:** 2006-11-20

**Authors:** Elsie M. Sunderland

**Affiliations:** U.S. Environmental Protection Agency, National Center for Environmental Research, Office of Research and Development, Boston, Massachusetts, USA

**Keywords:** Atlantic, fish imports, methylmercury, ocean, Pacific, per capita mercury intake, tuna

## Abstract

**Background:**

Methylmercury exposure causes a variety of adverse effects on human health. Per capita estimates of mercury exposure are critical for risk assessments and for developing effective risk management strategies.

**Objective:**

This study investigated the impact of natural stochasticity in mercury concentrations among fish and shellfish harvested from the Atlantic Ocean, Pacific Ocean, and foreign shores on estimated mercury exposures.

**Methods:**

Mercury concentrations and seafood consumption are grouped by supply region (Atlantic Ocean, Pacific Ocean, and foreign shores). Distributions of intakes from this study are compared with values obtained using national FDA (Food and Drug Administration) mercury survey data to assess the significance of geographic variability in mercury concentrations on exposure estimates.

**Results:**

Per capita mercury intake rates calculated using FDA mercury data differ significantly from those based on mercury concentration data for each supply area and intakes calculated for the 90th percentile of mercury concentrations.

**Conclusions:**

Differences in reported mercury concentrations can significantly affect per capita mercury intake estimates, pointing to the importance of spatially refined mercury concentration data. This analysis shows that national exposure estimates are most influenced by reported concentrations in imported tuna, swordfish, and shrimp; Pacific pollock; and Atlantic crabs. Collecting additional mercury concentration data for these seafood categories would improve the accuracy of national exposure estimates.

Human exposure to methylmercury (MeHg) causes a variety of adverse health effects, including developmental delays in children of exposed mothers (Cohen et al. 2005) and deficits in neurocognitive function in adults (Yokoo et al. 2003). Blood MeHg concentrations in individuals are strongly correlated with the frequency and types of seafood consumed (Mahaffey et al. 2004). However, even for pregnant women, consuming seafood has a variety of health benefits when dietary MeHg intake is known to be low (e.g., Daniels et al. 2004; Mozaffarian and Rimm 2006). Regulatory agencies rely on information about how individuals are exposed to MeHg to evaluate trade-offs among health benefits from fish consumption and potential risks of MeHg exposure.

In the United States, MeHg risk management takes the form of both advisories recommending limits on amounts of high-Hg fish consumed and regulations that control emissions from human sources. Assessing the effectiveness of both strategies in terms of changes in human exposure requires data on *a*) geographic supply regions for fish consumed by the U.S. population, and *b*) concentrations of Hg in fish and shellfish.

Comparing the supply of fisheries products for all individuals from the commercial market (18.9 g/person/day, 2000–2002) [National Marine Fisheries Service (NMFS) 2003] to the total intake from dietary recall surveys (16.9 g/person/day, uncooked fish weight, 1994–1996–1998) [U.S. Environmental Protection Agency (EPA) 2002] shows that mean consumption estimates are comparable in magnitude. Hence, across the entire U.S. population, most seafood consumed comes from the commercial market. Estuarine and marine fish and shellfish dominate the edible supply of fish in the commercial market, comprising > 90% of the market share (Carrington et al. 2004). Thus, dietary intake of MeHg from estuarine and marine seafood accounts for most exposure in the U.S. population.

Although many studies have investigated how variability in amounts and types of fish consumed affects MeHg exposure, few addressed uncertainties resulting from natural stochasticity in MeHg concentrations within seafood categories in the commercial market. Instead, most studies rely on Food and Drug Administration (FDA) survey data to characterize Hg concentration distributions (e.g., Carrington and Bolger 2002; Carrington et al. 2004; Mahaffey et al. 2004; Tran et al. 2004). However, FDA survey data are usually aggregated into one mean Hg concentration for each commercial market category. This can be problematic because each market category (e.g., fresh and frozen tuna) may describe a number of different biological species (e.g., for tuna: albacore, bigeye, bluefin, skipjack, yellowfin) with different growth rates and dietary preferences that affect Hg bioaccumulation. In addition, fish and shellfish in the commercial market consist of domestic landings from the Atlantic and Pacific oceans and imported species from a variety of countries.

Many researchers have reported geographic variability in Hg concentrations among commercially important fish and shellfish species. For example, various tuna species caught in the Atlantic, Pacific, and Mediterranean oceans have significantly different length- and weight-normalized tissue Hg residues (Adams 2004; Anderson and Depledge 1997; Brooks 2004; Morrisey et al. 2004; Storelli et al. 2002). In addition, although imported shrimp make up a large fraction of domestic seafood consumption (NMFS 2003), Hg concentrations reported by the FDA are typically below detection limits (FDA 2006a, 2006b). However, measured Hg concentrations in shrimp caught in a variety of countries vary by an order of magnitude (Minganti et al. 1996; Plessi et al. 2001; Ruelas-Izunza et al. 2004). Although high Hg concentrations can sometimes be attributed to sampling at contaminated sites (Chvojka et al. 1990) or age and size classes of fish not commonly found in the commercial seafood market, Burger et al. (2005) also found significant differences between nationwide FDA values and Hg levels in fish sold in seafood markets in the New Jersey region. Based on these data, we can hypothesize that variability in Hg intakes within each species category in the commercial market is not adequately captured by grouping Hg concentrations in fish caught in geographically diverse regions into a single population mean. Better resolution in Hg concentration data used for exposure assessments may be obtained by grouping survey data by the origin of each marine and estuarine seafood product in the commercial market.

This study assessed how estimated Hg exposure from estuarine and marine seafood in the U.S. population is affected by variability in Hg concentrations among different supply regions. To do this, supply of fisheries products were divided into categories based on the geographic sources of seafood in the commercial market consumed by the U.S. population. Expected Hg intake rates for different age groups, such as children and women of childbearing age, were modeled using Hg concentration data from each supply region, market share, and total consumption of each species from the NMFS (2001, 2002, 2003). Data from the U.S. Department of Agriculture’s Continuing Survey of Food Intake by Individuals (CSFII) (U.S. EPA 2002) and the National Health and Nutrition Examination Survey (NHANES) (NCHS 2006) provided information on variability in consumption patterns and body weights in the U.S. population. Distributions of intakes calculated in this study from geographically explicit Hg data were compared with values obtained using FDA Hg concentrations to assess whether variability in Hg concentrations by species and geographic regions significantly affects per capita intakes used to evaluate risks associated with Hg exposure. Geographically referenced exposure data provide a building block for quantitatively assessing how global changes in environmental Hg concentrations will affect human exposure to Hg in the United States.

## Methods

Species considered in this analysis comprise 77% of the total domestic landings reported by the NMFS for the years 2000–2002 and > 90% of the edible supply of fisheries products (NMFS 2001, 2002, 2003). The remaining domestic landings are freshwater species or are used for industrial purposes (i.e., fish oils, bait, animal meal).

Total dietary intake of Hg in the U.S. population from estuarine and marine fish and shellfish was modeled using data on the supply of fisheries products in the commercial market and their corresponding Hg concentrations. The supply of fisheries products is divided into four main categories, whereas Hg concentration data are split into three geographic designations. A fourth category was needed for supply because a portion of domestic landings (landings of fish and shellfish reported by domestic vessels) are actually harvested from the high seas (beyond the 200-mi exclusive economic zone marking U.S. waters) and at foreign ports. Hence, supply categories include *a*) Atlantic landings, *b*) Pacific landings, *c*) high seas and foreign ports landings, and *d*) imported seafood products that were not caught by U.S. vessels. Distributions of Hg concentration data for the Atlantic, Pacific, and “imported” seafood products were collected from a broad literature survey that included state and government databases (Table 1). Where primary data were available, distributions were fitted to the observed concentration values for different species. In cases where only means and SDs were reported, generic lognormal distributional forms were assumed, as in other studies (e.g., Carrington and Bolger 2002; Carrington et al. 2004).

### Supply of fisheries products

I used data on domestic landings, imports, exports, and re-exports reported by the NMFS (2001, 2002, 2003) to estimate the supply of fishery products from each region. All data were averaged over 3 years (2000–2002) to eliminate harvesting anomalies that might have occurred in an individual year. This study used NMFS data to estimate per capita consumption and to link each fisheries product back to its geographic origin. Annual consumption for the whole population, calculated using NMFS data, is also useful for inferring longer-term fish consumption trends that may not be captured by shorter dietary recall surveys such as NHANES (NCHS 2006) and CSFII (U.S. EPA 2002).

For each species considered, I calculated supply using information on domestic landings, imports, exports, and re-exports. To determine supply, exports were subtracted from edible weights of domestic landings, and re-exports (exports of imported products) were subtracted from imports. All landings were compiled for each individual species of fish or shellfish and then aggregated into commercial market categories, such as salmon, crab, shark, and tuna, that consist of multiple species. I converted domestic landings reported in live (whole fish) weights (NMFS 2006) to edible weights using information on the disposition of domestic landings (e.g., production of fillets and steaks, canned products, cured products) (NMFS 2001, 2002, 2003) and conversion factors for individual species and processed seafood products. Conversions of live weight to edible weight were obtained from ranges in edible yields for each fish species and seafood product reported by several data compilations [Crapo et al. 1993; Food and Agriculture Organization of the United Nations (FAO) 1989, 2004; Pacific Seafood Group 2006; Rick et al. 2002]. Although edible yields used in the present study represent averages or best estimates from these compilations, actual edible yields vary depending on factors such as condition of the fish and processing technique (Crapo et al. 1993; FAO 1989). Domestic landings were divided by ocean (Atlantic or Pacific) and by distance from shore. Distance from shore where harvest occurred provides data on quantity of fish caught in U.S. waters relative to those landed outside of the 200-mi exclusive economic zone (high seas) and foreign ports. I estimated market share (percent) from the total supply of estuarine and marine seafood for each category in the commercial market. Total supply of each fisheries product was scaled to match per capita consumption reported by the NMFS (2001, 2002, 2003), after accounting for the market share occupied by freshwater species based on Carrington et al. (2004). Results provide a total quantity of seafood consumed by the U.S. population for each source category (i.e., Atlantic, Pacific, high seas and foreign ports, and imports).

### Hg concentration data

I obtained information on the distribution of Hg concentrations in the commercial market from a variety of literature sources as well as from state and federal databases (Table 1). In cases where a variety of biological species are lumped into a single market category, Hg concentrations have been weighted by the fraction of landings of each species in each particular harvesting region. For example, reported domestic landings of 19 different species make up the commercial market category “crabs” (NMFS 2006). Based on total landings, important crab species in the commercial market are Atlantic rock (*Cancer irroratus*), blue (*Callinectes sapidus*), dungeness (*Cancer magister*), king (*Paralithodes camtschatica*), Florida snow claws (*Menippe mercenaria*), and snow/tanner (*Chinoecetes* spp.). Hg concentrations from Atlantic Ocean harvests were characterized using available data for the species harvested in that region (e.g., Atlantic rock, blue, and Florida stone claws) weighted by the portion of landings accounted for by each species. For some species (e.g., orange roughy, skate, tile-fish), no additional data other than FDA reported values (FDA 2006a, 2006b) were available (see Table 1 for details). In these cases, FDA data were used as a default. For comparative purposes between the present analysis and intakes calculated using FDA mean concentrations (FDA 2006a, 2006b), species reported as nondetects by the FDA were assigned a default value of 0.01 mg/kg. This default value was generally lower than Hg concentrations reported by other studies (Table 1).

One uncertainty in Hg concentration data for each species that has not been accounted for in this study is the fraction of total Hg present as MeHg in edible tissue (%MeHg). Although previous research by Bloom (1992) suggested that 95% of Hg in selected fish and invertebrates is MeHg, selected studies that have continued to measure MeHg in estuarine and marine species show considerable variability in %MeHg among different harvesting regions (e.g., Baeyens et al. 2003; Forsyth et al. 2004; Mason et al. 2006). Presently, data on %MeHg are insufficient to characterize regional variability among commercial species. Hence, I have not applied corrections for the fraction of total Hg present as MeHg.

### Statistical analyses and per capita intake estimates

For each species, variability in Hg concentrations reported in the literature was summarized using the mean ± SD and median of the observed data. I used Hg concentration distributions for each species as input values or uncertainties in the exposure model used to calculate total Hg intake for the population from estuarine and marine seafood. Supply of each seafood category was multiplied by the corresponding distribution of Hg concentrations using a Monte Carlo analysis to give percentiles of predicted Hg intakes. Intakes were then divided by the average U.S. population to calculate baseline per capita intake (micrograms of Hg per person per year).

I analyzed the sensitivity of model results (total Hg intake in the U.S. population) using Crystal Ball 7.2.1 (Decisioneering, Inc., Denver, CO) by ranking Hg distributions for each species by their importance (contribution to overall variance) in intake rates. Contributions to variance were calculated by squaring the rank correlation coefficients between every Hg concentration and every estimated intake and normalizing the results to 100%.

Differences between Hg concentrations and intakes for different supply regions and those based on FDA Hg data (FDA 2006a, 2006b) were analyzed for statistical significance using *t*-tests for paired means.

To extrapolate per capita Hg intakes to individual exposure, I used differences in fish consumption, body weights, and meal sizes from CSFII (U.S. EPA 2002) and NHANES (NCHS 2006) to compute scaling factors that account for demographic variability. Scaling factors were multiplied by the mean per capita Hg intake to allow for variability in fish consumption rates. Average body weights are based on NHANES survey data for 1999–2002 (NCHS 2006). Resulting variability in Hg intake estimates for each demographic group (micrograms of Hg per kilogram body weight per day) facilitates comparison with the U.S. EPA reference dose (RfD) [National Research Council (NRC) 2000] for MeHg and the potential for adverse health effects in the population.

## Results

**Figure 1:**
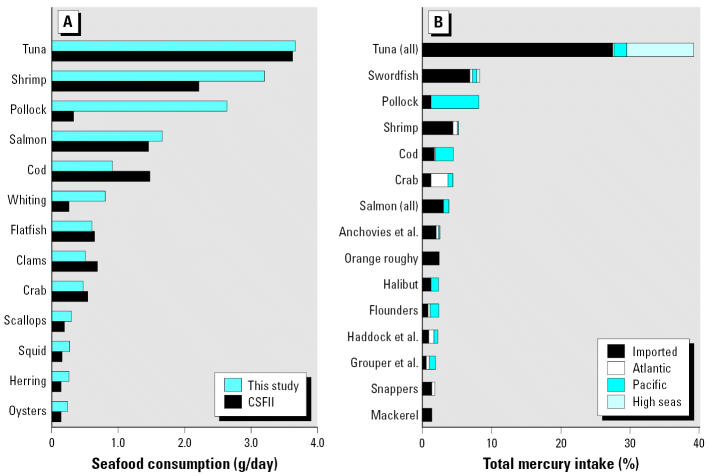
Seafood consumption and total Hg intake from estuarine and marine fish and shellfish in the commercial market. (*A*) Seafood consumption estimated in this study from NMFS fisheries supply data compared with available data for marine and estuarine fish consumption from CSFII dietary survey data [uncooked weights (U.S. EPA 2002]. (*B*) Percentage of total Hg intake (product of seafood supply and Hg concentrations) for the top 15 seafood categories; intake is allocated by the source region for each of the fisheries products [Atlantic, Pacific, imported (foreign sources), and high seas landings]. “Salmon” includes both canned and fresh and frozen products; “Anchovies et al.” includes anchovies, herring, shad, and sardines; “Flounders” includes flounder, plaice, and sole; “Haddock et al.” includes haddock, hake, whiting, and monkfish; and “Grouper et al.” includes grouper and seabass (Table 1).

Differences between seafood consumption rates calculated in the present study using NMFS data (NMFS 2001, 2002, 2003) and intake data from CSFII (uncooked weight, all individuals) (U.S. EPA 2002) shown in Figure 1A are relatively small (relative error of absolute differences < 3%). These results indicate that NMFS data compiled in this study provide a reasonable inventory of fish consumption for all individuals in the United States. Differences are most pronounced for estimated pollock consumption. However, this variability may be explained in part by greater uncertainty among participants identifying highly processed products such as fish sticks and imitation meats, which are frequently pollock.

For estuarine and marine species, tuna are the dominant source of Hg intake across the entire U.S. population, accounting for 39% of total intake calculated from Hg concentration data compiled in the present study and 43% using FDA Hg concentration data (FDA 2006a, 2006b) (Figure 1B). Intake from tuna products in this category includes fresh and frozen tuna (11%), canned light tuna (18%), and canned albacore/white tuna (10%). Other significant sources of Hg include swordfish (8%), pollock (8%), shrimp (5%), and cod (4.5%).

**Figure 2:**
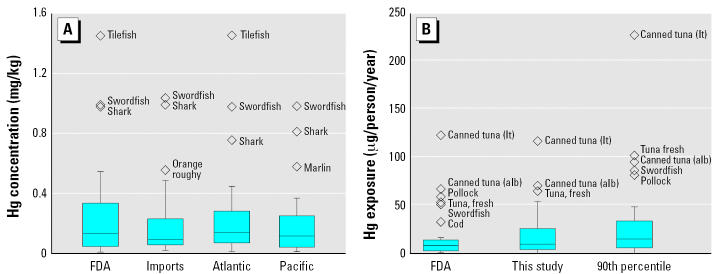
Summary of Hg concentrations (*A*) and Hg intakes (*B*) for all estuarine and marine seafood categories in the commercial market compared with FDA concentrations and intakes calculated from FDA data (Table 1). Abbreviations: alb, albacore/white; lt, light. The bottom and top of each box indicate 25th and 75th percentiles, respectively; the line within the box indicates the median; and whiskers indicate minimum and maximum. Outliers (any point falling above the upper quartile minus 1.5 times the interquartile range) are shown above the plots. In (*B*), intakes calculated from geographically grouped data are denoted “This Study” for mean per capita intakes and “90th percentile” for intakes calculated from the 90th percentile mercury concentrations for each geographic region.

When Hg data for each supply region (imported, Atlantic, Pacific) and the FDA (Table 1) are condensed into a single population, the median values (Figure 2A) and mean of means from each sample set are comparable in magnitude. Accordingly, statistical tests show that overall mean Hg concentrations for each supply region do not differ significantly from FDA (Table 1) values (*p* > 0.05, *t*-test, paired means). In contrast, Figure 2B shows the statistically significant differences between Hg intake rates calculated using Hg concentration data for each supply area and those based on FDA Hg data (Table 1) (*p* < 0.05, *t*-test, paired means). These statistical differences are even greater when comparing per capita intake based on FDA Hg data to intake calculated using the 90th percentile Hg concentrations for species from each geographic region (*p <* 0.01, *t*-test, paired means). Seafood categories with no geographically specific Hg data other than FDA values were excluded from this sample comparison.

Model sensitivity analysis shows that variability in Hg concentrations in imported canned light tuna has the greatest relative effect on variance in forecasted total Hg intake. Summed over all seafood categories and for all geographic regions, modeled intake rates are most sensitive to variability in Hg concentrations in imported canned light tuna (64% of the total variance), followed by imported fresh and frozen tuna (11%), imported swordfish (7%), Pacific pollock (6%), imported canned albacore tuna (5%), Atlantic crab (3%), and imported shrimp (1%). Variability in Hg concentrations in remaining seafood categories accounts for the remaining variance in intakes.

## Discussion

### Population-wide Hg intake

Results for population-wide Hg intakes from different seafood categories (Figure 1B) are generally consistent with estimates from other studies showing the dominant role of both frequently consumed species, such as canned tuna and pollock, and high Hg species such as swordfish (Carrington and Bolger 2002; Carrington et al. 2004) on overall exposures. When considering trade-offs among potential risks and benefits from seafood consumption (Mozaffarian and Rimm 2006), it is useful to note that most species, regardless of geographic origin, are fairly low in Hg (0.10–0.15 mg/kg) and contribute relatively small amounts to Hg exposure in the U.S. population (Figure 2). Model sensitivity analysis indicates that collecting additional monitoring data for tuna species common in the commercial market, as well as swordfish, shrimp, Pacific pollock, and Atlantic crabs, would result in the greatest improvements in per capita exposure estimates.

In particular, additional data on differences in tuna concentrations among global harvest regions are needed to improve the reliability of Hg exposure estimates for public health protection. Using average market sizes of tuna to normalize measured Hg concentrations constrains concentrations to ranges most likely to be found in the market and consumed (Table 2). For example, published regression relationships for albacore tuna (*Thunnus alalunga*) show that for the average market size (12 kg), concentrations in tuna from the Mediterranean Sea (0.87 mg/kg) are higher than those in the Atlantic (0.47 mg/kg) and Pacific (0.17 mg/kg) oceans (Table 2). This is not unexpected because the Mediterranean is naturally enriched in cinnabar deposits (Bacci 1989), and total and methyl Hg concentrations in subsurface ocean water appear to be higher than in the Atlantic or North Pacific (Mason and Gill 2005). Preliminary data for bluefin and yellowfin tuna are consistent with trends observed for albacore tuna (Table 3). However, few data describing the geographic origin or species composition of tuna in canned products are available, making it difficult to establish a relationship between Hg concentrations in live tuna and those in canned tuna consumed in the United States. Additional Hg concentration data resolved by harvest region for tuna should be a priority for future study, given the importance of variability in tuna concentrations, especially canned products, on overall Hg exposure levels.

### Per capita Hg intakes and individual exposure

Having established that geographic variability of Hg concentrations in different species affects per capita intakes, one naturally desires a further analysis incorporating variability in quantities of seafood and selections of species chosen by individuals. Unfortunately, available survey data [NHANES, CSFII (NCHS 2006; U.S. EPA 2002)] do not yet include the geographic origin of fish consumed. Thus, dietary survey data alone do not enable a combined analysis of geographic variability and individual diet choices of species. However, using NMFS data compiled in the present study, one can combine geographic variability of Hg concentrations with individual choices of seafood quantity. This partially accounts for observed differences between nationwide averages and fish consumption among populations susceptible to Hg exposure (Moya 2004).

To explore Hg intakes among high fish consumers, the combined NMFS and CSFII data (e.g., NCHS 2006; U.S. EPA 2002) were applied to predict per capita Hg intakes at various quantities of fish consumed. Although it reflects a population average, market share occupied by each species (NMFS 2001, 2002, 2003) provides a proxy for individual diet selection (Figure 1A). In Table 3, the rows reflect percentiles of exposures based on seafood Hg levels that vary both geographically and across species. The columns reflect variability in exposures as a function of the quantity of seafood consumed by different demographic groups. Table 3 shows that, at the 90th percentile consumption rate, exposures based on fish Hg means reported by the FDA (Table 1) would suggest that any individual selecting this proxy diet would be exposed to Hg at levels below the U.S. EPA RfD (NRC 2000). However, exposures based on geographic variability in fish Hg suggest that a fraction of each demographic group will exceed the U.S. EPA RfD.

To explore the impact of assuming this proxy diet, these results can be compared with exposure assessments that incorporate information on diet selection variability. Relying only on dietary survey data and fish Hg averages, a complementary analysis performed by Tran et al. (2004) showed exposures for children and women of childbearing age. Their resulting 90th and 95th percentile exposures, 0.12 and 0.20 μg/kg/day, respectively, fall within the ranges of exposure predicted by this study for 90th and 95th percentile fish consumers (0.07–0.29 and 0.11–0.46 μg/kg/day, respectively). These ranges result from considering geographic variability in fish Hg concentrations. To go beyond the present analysis, one would need intake estimates that combine fish harvest region with consumption quantities and species selection. Variability in fish Hg concentrations may help to explain differences in mean and 90th percentile blood Hg concentrations observed for Atlantic coastal residents (2.7 and 7.7 μg/L, respectively) relative to those measured in Pacific coastal residents (1.7 and 4.7 μg/L, respectively) (Mahaffey 2005).

Table 4 shows the impact of variations in fish Hg across harvest regions on estimated exposures for women of childbearing age as a function of meal frequency (NHANES 1999–2000) and meal size (CSFII 1994–1998) (Mahaffey et al. 2004; U.S. EPA 2002). Women of childbearing age and average weight (73 kg) consuming more than eight large fish meals (> 315 g/meal) per month are expected to exceed the RfD. However, even at more than eight meals per month, consuming average portion sizes (115 g/meal) results in a distribution of exposures in which all but the 99th percentile are below the RfD. These results generally agree with empirical data on blood Hg levels for 1999–2002, showing that approximately 6% of U.S. women of child-bearing age (3.8 million individuals) exceed the U.S. EPA RfD for MeHg (Jones et al. 2004). Geographic variability in fish Hg merits consideration in future efforts to understand elevated blood Hg levels in human populations.

## Figures and Tables

**Table 1:** Hg concentration data (mg/kg) aggregated by geographic region.

Species	FDA (mean ± SD)	No.	References	Imports (mean ± SD)	No.	References	Atlantic (mean ± SD)	No.	References	Pacific (mean ± SD)	No.	References
Anchovies	0.04	40	NMFS 1978	0.06 ± 0.01	53	Burger et al. 2005; Capelli et al. 2004; Knowles et al. 2003	No landings			0.04 ± 0.01	40	NMFS 1978
Herring	0.04	38	NMFS 1978	0.13 ± 0.03	14	Baeyens et al. 2003; Legrand et al. 2005; Nakagawa et al. 1997	0.14 ± 0.06	15	U.S. EPA 2003	0.04 ± 0.02	131	U.S. EPA 2003
Sardine	0.02	22	FDA 2006a	0.03 ± 0.003	35	Knowles et al. 2003; Nakagawa et al. 1997; Plessi et al. 2001	No landings			No landings		
Shad	0.07	59	NMFS 1978	0.07 ± 0.01	59	NMFS 1978	0.02 ± 0.02	40	U.S. EPA 2003	0.07 ± 0.01	59	NMFS 1978
Bluefish	0.34 ± 0.13	52	FDA 2006a	None consumed			0.45 ± 0.33	288	U.S. EPA 2003	No landings		
Clams^a^	ND	6	FDA 2006a	0.06 ± 0.01	3	Plessi et al. 2001	0.01 ± 0.002	4	Legrand et al. 2005	0.01 ± 0.002	2	U.S. EPA 2003
Cod	0.10 ± 0.08	39	FDA 2006a	0.07 ± 0.01	19	Baeyens et al. 2003; Nakagawa et al. 1997; Plessi et al. 2001	0.06 ± 0.02	21	Gobeil et al. 1997; Legrand et al. 2005	0.11 ± 0.03	28	U.S. EPA 2003
Crabs	0.06 ± 0.11	63	FDA 2006a	0.10 ± 0.02	27	Dabeka et al. 2004; Legrand et al. 2005; Plessi et al. 2001	0.26 ± 0.44	369	U.S. EPA 2003	0.15 ± 0.07	56	Dabeka et al. 2004; Bloom 1992; U.S. EPA 2003; Hui et al. 2005
Croaker	0.07 ± 0.04	50	FDA 2006a	None consumed			0.07 ± 0.08	315	U.S. EPA 2003	0.12 ± 0.10	45	U.S. EPA 2003
Haddock	0.03 ± 0.02	4	FDA 2006a	0.06 ± 0.01	31	Joiris et al. 1995; Legrand et al. 2005	0.03 ± 0.02	4	FDA 2006a	No landings		
Hake and whiting^b^	0.01 ± 0.02	11	FDA 2006a	0.13 ± 0.01	88	Baeyens et al. 2003; Capelli et al. 2004; Plessi et al. 2001	0.07 ± 0.02	22	Burger et al. 2005; U.S. EPA 2003	0.01 ± 0.02	11	FDA 2006a
Monkfish	0.18	81	NMFS 1978	0.13 ± 0.01	25	Baeyens et al. 2003; Knowles et al. 2003; Plessi et al. 2001	0.18 ± 0.04	81	NMFS 1978	No landings		
Flounder^c^	0.05 ± 0.05	23	FDA 2006a	0.05 ± 0.07	55	Burger et al. 2005	0.08 ± 0.04	60	U.S. EPA 2003	0.07 ± 0.07	58	Burger et al. 2005; U.S. EPA 2003
Plaice^c^	0.05 ± 0.05	23	FDA 2006a	0.05 ± 0.02	33	Baeyens et al. 2003	0.05 ± 0.02	33	Baeyens et al. 2003	No landings		
Sole^c^	0.05 ± 0.05	23	FDA 2006a	0.10 ± 0.10	64	Baeyens et al. 2003; Plessi et al. 2001	No landings			0.06 ± 0.02	518	U.S. EPA 2003
Grouper	0.47 ± 0.29	43	FDA 2006a	0.34 ± 0.07	17	Al-Saleh and Al-Doush 2002; Knobeloch et al. 1995	0.36 ± 0.14	100	U.S. EPA 2003	0.47 ± 0.29	43	FDA 2006a
Sea bass	0.22 ± 0.23	47	FDA 2006a	0.19 ± 0.12	29	Baeyens et al. 2003; Knowles et al. 2003; Legrand et al. 2005; Nakagawa et al. 1997	0.14 ± 0.04	14	U.S. EPA 2003	0.22 ± 0.23	47	FDA 2006a
Rockfish^d^	0.22 ± 0.23	47	FDA 2006a	None consumed			No landings			0.29 ± 0.22	314	U.S. EPA 2003
Halibut	0.25 ± 0.23	46	FDA 2006a	0.23 ± 0.05	11	Knowles et al. 2003; Plessi et al. 2001	0.25 ± 0.23	46	FDA 2006a	0.28 ± 0.09	11	U.S. EPA 2003
Scorpionfish^e^	0.29	78	NMFS 1978	0.11 ± 0.003	7	Nakagawa et al. 1997; Plessi et al. 2001	No landings			0.22 ± 0.05	79	Bloom 1992; NMFS 1978
Lobster	0.17 ± 0.09	16	FDA 2006a	0.10 ± 0.005	13	Knowles et al. 2003; Legrand et al. 2005; Plessi et al. 2001	0.28 ± 0.15	106	NMFS 1978; U.S. EPA 2003	0.17 ± 0.09	16	FDA 2006a
Mackerel, all^f^	0.15	432	NMFS 1978; U.S. EPA 2000	0.15 ± 0.10	432	NMFS 1978; U.S. EPA 2000	0.22 ± 0.16	877	NMFS 1978; U.S. EPA 2003	0.09 ± 0.06	30	NMFS 1978; U.S. EPA 2000
Marlin^a^	0.49 ± 0.24	16	FDA 2006a	0.49 ± 0.24	16	FDA 2006a	No landings			0.57 ± 0.41	39	Brooks 2004
Mussels^g^	NA	NA	NA	0.03 ± 0.009	80	Baeyens et al. 2003; Dabeka et al. 2004; Knowles et al. 2003; Plessi et al. 2001	0.08 ± 0.09	729	U.S. EPA 2003	0.03 ± 0.02	330	U.S. EPA 2003
Oysters	ND	34	FDA 2006a	0.01 ± 0.01	27	Dabeka et al. 2004	0.07 ± 0.09	2,082	U.S. EPA 2003	0.06 ± 0.03	63	U.S. EPA 2003
Ocean perch	ND	6	FDA 2006a	0.09 ± 0.02	53	Joiris et al. 1995; Plessi et al. 2001	0.08 ± 0.02	50	Joiris et al. 1995	0.08 ± 0.02	50	Joiris et al. 1995
Orange roughy	0.54	26	FDA 2006a	0.55 ± 0.11	32	FDA 2006a; Knowles et al. 2003	No landings			No landings		
Pollock	0.06	37	FDA 2006a	0.03 ± 0.002	12	Knowles et al. 2003; Legrand et al. 2005; Plessi et al. 2001	0.02 ± 0.01	115	U.S. EPA 2003	0.06 ± 0.03	37	FDA 2006a
Sablefish	0.22	102	NMFS 1978	0.22 ± 0.04	102	NMFS 1978	No landings			0.22 ± 0.04	103	Bloom 1992; FDA 2006a
Salmon, fresh	0.01	34	FDA 2006a	0.04 ± 0.01	69	FDA 2005; Dabeka et al. 2004; Knowles et al. 2003; Legrand et al. 2005; Plessi et al. 2001	0.13 ± 0.17	11	U.S. EPA 2003	0.04 ± 0.01	289	U.S. EPA 2003
Salmon, canned	ND	34	FDA 2006a	0.04 ± 0.01	32	Knowles et al. 2003	No landings			0.04 ± 0.01	289	U.S. EPA 2003
Scallops	0.05	66	NMFS 1978	0.06 ± 0.02	21	Legrand et al. 2005; Nakagawa et al. 1997	0.01 ± 0.003	12	Burger et al. 2005	0.04 ± 0.001	3	Bloom 1992
Sea trout	0.25	27	FDA 2006a	None consumed			0.21 ± 0.15	1,220	U.S. EPA 2003	No landings		
Shrimp	ND	24	FDA 2006a	0.03 ± 0.01	106	Al-Saleh and Al-Doush 2002; Burger et al. 2005; Dabeka et al. 2004; FDA 2005; Plessi et al. 2001	0.04 ± 0.05	171	U.S. EPA 2003	0.03 ± 0.01	44	FDA 2005
Skate	0.14	56	NMFS 1978	None consumed			0.14 ± 0.03	56	NMFS 1978	0.14 ± 0.03	56	NMFS 1978
Snapper	0.19 ± 0.12	25	FDA 2006a	0.21 ± 0.15	324	Burger et al. 2005; Chvojka et al. 1990	0.28 ± 0.43	363	U.S. EPA 2003	0.25 ± 0.09	17	U.S. EPA 2003
Porgy	NA	NA	NA	None consumed			0.08 ± 0.07	14	U.S. EPA 2003	No landings		
Sheepshead	0.13	59	NMFS 1978	None consumed			0.18 ± 0.20	268	U.S. EPA 2003	No landings		
Squid	0.07	200	NMFS 1978	0.07 ± 0.01	200	NMFS 1978	No supply			No supply		
Shark	0.99 ± 0.63	351	FDA 2006a	0.99 ± 0.63	351	FDA 2006a	0.75 ± 0.70	585	U.S. EPA 2003	0.80 ± 0.37	35	U.S. EPA 2003
Swordfish	0.98 ± 0.51	618	FDA 2006a	1.03 ± 0.54	689	Bloom 1992; Dabeka et al. 2004; FDA 2006a; Knowles et al. 2003; Nakagawa et al. 1997; Plessi et al. 2001	0.98 ± 0.51	618	FDA 2006a	0.98 ± 0.51	618	FDA 2006a
Tilefish	1.45	60	NMFS 1978	None consumed			1.45 ± 0.29	60	NMFS 1978	No landings		
Tuna, canned albacore	0.35	179	FDA 2006b	0.37 ± 0.12	318	Burger and Gochfeld 2004; FDA 2006b; Forsyth et al. 2004	0.37 ± 0.12	318	Burger and Gochfeld 2004; FDA 2006b; Forsyth et al. 2004	0.37 ± 0.12	318	Burger and Gochfeld 2004; FDA 2006b; Forsyth et al. 2004;
Tuna, canned light	0.12	131	FDA 2006b	0.11 ± 0.10	199	Burger and Gochfeld 2004; Dabeka et al. 2004; FDA 2006b	0.11 ± 0.10	199	Burger and Gochfeld 2004; Dabeka et al. 2004; FDA 2006b	0.11 ± 0.10	199	Burger and Gochfeld 2004; Dabeka et al. 2004; FDA 2006b
Tuna, fresh and frozen	0.38	131	FDA 2006b	0.48 ± 0.24	422	Burger et al. 2005; Dabeka et al. 2004; FDA 2006b; Harding et al. 2005; Storelli et al. 2002; Storelli and Marcotrigiano 2004	0.28 ± 0.12	496	Adams 2004; Anderson and Depledge 1997; FDA 2006b; Harding et al. 2005; U.S. EPA 2003	0.24 ± 0.10	555	Brooks 2004; FDA 2006b; Morrissey et al. 2004
Whitefish	0.07 ± 0.05	25	FDA 2006a	0.07 ± 0.01	25	FDA 2006a	No landings			No landings		

Abbreviations: NA, not applicable; ND, below detection limits. For comparative analysis, FDA nondetects were assigned a default value of 0.01 mg/kg. All FDA data are from FDA (2006a, 2006b).

a FDA measured as methylmercury.

b Whiting listed as below detection limits by FDA (*n* = 2); hake values were used for comparative analysis.

c Listed by FDA as flatfish, which includes flounder, plaice, and sole.

d Includes seabass, striped bass, and rockfish.

e Includes lingcod.

f Mackerel concentrations for all species calculated by weighting Hg concentrations by percent domestic landings for each species: king (8%), Spanish (6%), Atlantic (47%), chub (39%).

g No concentrations reported by FDA; the default of 0.01 mg/kg was used for comparative analysis.

**Table 2:** Summary statistics for all tuna species in the U.S. commercial seafood market.

					Hg (mg/kg) global harvest (%)^a^		
Species	Market size^a^	Fresh (%)^b^	Domestic waters (%)^c^	Products	Pacific	Atl/Med^d^	Indian
Albacore (*Thunnus alalunga*)	9–20 kg, 68 cm	9	< 1	Canned (white) and fresh/frozen	0.17 (67)	Atl 0.47/Med 0.87 (25)	(8)
Bigeye (*Thunnus obesus*)	15–20 kg, 90 cm	13	34	Fresh/frozen	0.29 (60)	(25)	(15)
Bluefin (*Thunnus thynnus*)	~ 7 kg	2	38	Canned (white) and fresh/frozen	(40)	0.13^e^ (60)	(0)
Skipjack (*Katsuwonus pelamis*)	~ 3 kg, 35 cm	38	1	Canned (light) and fresh, smoked	(67)	0.17 (13)	(20)
Yellowfin (*Thunnus albacares*)	5–20 kg, 40–180 cm	34	7	Canned (light) and fresh, smoked	0.06 (60)	Atl 0.31 (15)	(25)

Abbreviations: Atl, Atlantic; Med, Mediterranean. Hg concentrations are for average market size of each species calculated from regression relationships published in the literature: data for Pacific albacore tuna from Morrissey et al. (2004); Pacific yellowfin and bigeye data from Brooks (2004); Mediterranean albacore and bluefin data from Storelli et al. (2002); Atlantic albacore and bluefin data from Anderson and Depledge (1997); Atlantic yellowfin data from Adams (2004); and Atlantic bluefin data from Harding et al. (2005).

a Data from Atuna (2006).

b Fraction of fresh and frozen tuna sold in the U.S. commercial seafood market by species; species other than those listed account for 4% of the supply.

c Estimated fraction of supply of fresh and frozen tuna for each species that is caught in domestic waters in the U.S. (within the 200-mi exclusive economic zone).

d Atlantic and Mediterranean tuna are merged into a single data set because they do not appear to be significantly different once normalized to weight. This may be an attribute of the highly migratory nature of bluefin tuna; therefore, harvest areas do not necessarily reflect a dominant habitat for bluefin tuna (Block et al. 2001).

**Table 3:** Effect of variability in Hg concentrations and seafood consumption rates (percentiles) on Hg intakes (μg/kg body weight/day).

	Demographic group		Estimated Hg intake (percentiles based on fish Hg concentration variability)^a^						
Seafood consumption^b^	Sex, age (years)	Avgerage bw (kg)^c^	Mean FDA	Mean	50th	75th	90th	95th	99th
Per capita	All individuals	68.9	0.02	0.02	0.02	0.03	0.03	0.03	0.09
50th	F and M, ≤ 14	33.7	0.02	0.02	0.02	0.03	0.03	0.03	0.09
50th	F, 15–44	72.6	0.02	0.02	0.02	0.02	0.03	0.03	0.08
50th	M, 15–44	84.4	0.02	0.02	0.02	0.03	0.03	0.03	0.09
50th	≥ 45	80.2	0.03	0.03	0.03	0.03	0.04	0.04	0.11^d^
90th	≤ 14	33.7	0.07	0.08	0.08	0.08	0.09	0.10^d^	0.29^d^
90th	F, 15–44	72.6	0.07	0.08	0.07	0.08	0.09	0.10^d^	0.29^d^
90th	M, 15–44	84.4	0.08	0.09	0.09	0.10^d^	0.11^d^	0.12^d^	0.35^d^
90th	≥ 45	80.2	0.09	0.10^d^	0.10^d^	0.11^d^	0.12^d^	0.14^d^	0.38^d^
95th	≤ 14	33.7	0.13^d^	0.15^d^	0.15^d^	0.16^d^	0.19^d^	0.20^d^	0.57^d^
95th	F, 15–44	72.6	0.11^d^	0.12^d^	0.12^d^	0.13^d^	0.15^d^	0.16^d^	0.46^d^
95th	M, 15–44	84.4	0.13^d^	0.14^d^	0.14^d^	0.15^d^	0.17^d^	0.19^d^	0.53^d^
95th	F and M,≥ 45	80.2	0.13^d^	0.15^d^	0.15^d^	0.16^d^	0.18^d^	0.20^d^	0.57^d^

Abbreviations: bw, body weight; F, female; M, male.

a Exposures are calculated assuming species composition matches relative supply in the commercial seafood market; variability in measured Hg concentrations for each geographic region (imported, Atlantic, Pacific) and within each species was modeled using 10^5^ Monte Carlo trials.

b Modeled based on variability in CSFII data (1994–1996–1998) for each age group (U.S. EPA 2002).

c Based on NHANES survey data 1999–2002 (NCHS 2006).

d Intake rates that exceed the U.S. EPA RfD for MeHg (NRC 2000).

**Table 4:** Modeled effects (mean and percentiles) of variability in Hg concentrations on potential exposure for women of childbearing age.

		Modeled distribution of Hg intake (μg/kg bw/day)^a^						
Fish meals/month^b^^,^^c^	Meal size (percentile)^c^^,^^d^	Mean FDA	Mean	50th	75th	90th	95th	99th
1–4 (46)	10th (5)	< 0.01	< 0.01	< 0.01	< 0.01	< 0.01	< 0.01	0.01–0.03
	50th (23)	0.01–0.02	0.01–0.03	0.01–0.03	0.01–0.03	0.01–0.04	0.01–0.04	0.03–0.11
	90th (5)	0.02–0.07	0.02–0.08	0.02–0.08	0.02–0.08	0.02–0.09	*0.03–0.10*	*0.07–0.29*
5–8 (13.5)	10th (1)	0.01–0.02	0.01–0.02	0.01–0.02	0.01–0.02	0.01–0.02	0.01–0.02	0.04–0.06
	50th (7)	0.03–0.05	0.04–0.06	0.03–0.06	0.04–0.06	0.04–0.07	0.05–0.08	*0.13–0.21*
	90th (1)	*0.09–0.14*	*0.10–0.16*	*0.10–0.15*	*0.10–0.17*	*0.12–0.19*	*0.13–0.21*	*0.36–0.58*
> 8 (9)	10th (< 1)	> 0.02	> 0.02	> 0.02	> 0.02	> 0.02	> 0.03	> 0.07
	50th (4.5)	> 0.06	> 0.06	> 0.06	> 0.07	> 0.08	> 0.09	> *0.24*
	90th (< 1)	> *0.15*	> *0.18*	> *0.17*	> *0.20*	> *0.21*	> *0.23*	> *0.66*

Abbreviations: bw, body weight; Women of childbearing age are defined as being 15–44 years of age in the CSFII and 16–49 years of age in NHANES. All exposures above the U.S. EPA RfD (NRC 2000) are shown in italics.

a Intakes are calculated from average body weights from NHANES data (NCHS 2006).

b NHANES 1999–2000 data are from Mahaffey et al. (2004).

c The percent of total respondents (*n* = 1,707) consuming fish at varying frequencies over 30-day period is shown in parentheses; individuals who reported no fish consumption are not shown.

d Data from Tran et al. (2004) for all fish consumption by women of childbearing age from CSFII data between 1994 and 1998; based on survey data, meal sizes are as follows: 10th percentile = 33.6 g; mean = 115.3 g; 90th percentile = 315.2 g.
